# Further validation of the Health Scale of Traditional Chinese Medicine (HSTCM)

**DOI:** 10.1186/1749-8546-4-8

**Published:** 2009-04-30

**Authors:** Darong Wu, Shilong Lai, Luojing Zhou, Xinfeng Guo, Weixiong Liang, Zehuai Wen, Aihua Ou, Guangqing Zhang, Keji Chen

**Affiliations:** 1 Second Affiliated Hospital of the Guangzhou University of Chinese Medicine, Guangzhou, PR China; 2 Guangzhou University of Chinese Medicine, Guangzhou, PR China; 3 Xiyuan Hospital, China Academy of Chinese Medical Sciences, Beijing, PR China

## Abstract

**Background:**

Few health measurement scales are based on Chinese medicine theory. The Health Scale of Traditional Chinese Medicine (HSTCM) was developed to fill this gap. The aim of this study is to validate the HSTCM.

**Methods:**

A convenience sample of 630 participants was recruited in 11 settings. All participants were asked to complete the HSTCM and World Health Organization Quality of Life Measure-Abbreviated Version (WHOQOL-BREF).

**Results:**

Properties of the HSTCM were tested. Intra-class correlation coefficient representing the inter-interviewer reliability was 0.99 (95%CI) for the overall instrument. Spearman-Brown correlation coefficient and Cronbach's coefficient alpha were 0.81 and 0.94 respectively, indicating satisfactory internal reliability and inter-interviewer reliability. Spearman's rho correlation coefficient between the HSTCM and WHOQOL-BREFF was -0.67. A receiver operating characteristic (ROC) curve analysis was performed to test the discriminate validation. Areas under the ROC curve analysis for the HSTCM and its domains ranged 0.71–0.87 and all the lower levels of 95%CI were greater than 0.50.

**Conclusion:**

The HSTCM was validated as a generic health scale and may complement existing health measurement scales in Chinese medicine health care.

## Background

Acceptance of health measurement scales based on one's perception has increased in recent years [1-3]. Several well-established measures on health status or health-related Quality of Life (QOL) have been widely used [4-8]; however, few were developed on the basis of Chinese medicine which has been important for the well-being of the Chinese people for many years [9] and is now increasingly recognized worldwide [10-12].

Measuring health in accordance with Chinese medicine theory is useful [13,14]. Chinese medicine practitioners pay close attention to patients' subjective feelings for diagnosis and treatment. Different perspectives of the patients may lead to different prescriptions from the practitioner even if the patients suffer from the same condition [15]. Over the years, Chinese medicine practitioners have accumulated valuable experiences on obtaining information from the patient's perspective that may benefit their treatment strategy and judgment of clinical efficacy. We believe that these experiences can be more easily understood by clinicians and researchers if they were involved in some applicable instruments. Moreover, Chinese medicine and Western medicine work differently towards the end result. In Chinese medicine, information such as adaptation to climates and seasons, aversion to cold, positive feelings concerning the ability of maintaining stable mood is carefully captured as evidence for patients' adaptability with both natural and social environments. Although modern Chinese medicine practitioners may take biomarkers as references, there is a general impression among them that indicators reflect individual's adaptability may possess a more important position when testing the efficacy of a Chinese medicine treatment.

However, these indicators are usually not involved in common patient reported outcomes (PRO) or health related quality of life measures. A few other instruments were developed on the basis of Chinese medicine theory or for integrative medical research but none of them were based on a community sample [16,17]. In addition, the commonly recognized and accepted set of standardized procedures [18,19], from concept to measurement scale, makes it possible to develop an instrument based on Chinese medicine theory.

The Health Scale of Traditional Chinese Medicine (HSTCM), which is a generic scale designed for evaluating general health according to subjective feelings [20], is based on health concept of Chinese medicine and the operationalization of this concept's measurement [20,21]. The HSTCM consists of three domains, namely physical function under natural environment (PFNE), spirit (SP) and social environment (SE), and 2 additional items (see Additional file 1). PFNE domain has four facets: physical functioning (PF), voice (VOC), stool and urine (SU), and adaptability of natural environment (ANE). SP domain also has four facets: confidence and content (CC), self-confidence (SC), energy (EG) and basic ability of thinking (BAT). SE domain has three facets: ability of communicating with people (ACP), adaptability of noisy condition (ANC) and ability of dealing with bad stimulations (ADBS). In 2003, the development of initial Health Scale of Traditional Chinese Medicine (iHSTCM) [20] and the refinement from the iHSTCM (88 items) to HSTCM (47 items) [21] were accomplished. It was shown that the HSTCM was conceptually sound and reliable [22]. However, the test of psychometric properties using various independent samples may still be needed, particularly construct validation, according to the principles of instrument development [23,24]. Meanwhile, the previous test was based on data collected through iHSTCM (containing 88 items [21,22]); it is reasonable to test the psychometric properties again with the revised version of HSTCM (containing 47 items).

The purpose of developing the HSTCM is similar to other generic health related QOL measures, e.g. the World Health Organization Quality of Life Measure-Abbreviated Version (WHOQOL-BREF) [25] and MOS 36-Item Short Form (SF-36) [6]. We used the WHOQOL-BREF (Chinese version) [26] as a comparison instrument when examining the validation of the HSTCM because of the excellent validity and reliability of the WHOQOL-BREF. It is also because the Chinese version of the WHOQOL-BREF has become one of the standard measurements of QOL in China.

The aim of this study is to validate the measurement properties of the HSTCM in a convenience sample.

## Methods

### Participants

Inclusion criteria for the participants are (a) 18 years of age or above and (b) speaking *Putonghua *(Mandarin Chinese). All participants were asked to complete both the HSTCM and WHOQOL-BREF. An oral agreement was obtained from each participant prior to interview.

### Study instruments

The HSTCM includes 47 items, each with a five-degree response format, scoring 1, 2, 3, 4 and 5 respectively. Total scores range 0–225 (two additional items not included), whereby a high score indicates poor health. The Chinese version of the WHOQOL-BREF (Ministry of Health, China, 1999) [26] was taken in this study as a standard criterion. The instrument contains 26 core items and three additional items. The three additional items are only used in the mainland Chinese version.

### Demographics

A demographic questionnaire covering gender, age, and highest educational attainment was provided at the end of the interview. Each participant completed the questionnaire in the presence of an interviewer.

### Quality control of data collection

We produced a booklet entitled the *Guide to the HSTCM Survey *to set forth a standard operating procedure (SOP), rules of investigation, structured response to frequently asked questions for interviewers and structured oral interview guidance. A total of 25 interviewers, who were senior medical students or nurses, were trained with the booklet for 4–6 hours.

### Data management and analysis

We compiled a coding notebook to ensure good quality of data entry. If there are two answers to one question that are next to each other, the auditor must choose one of the answers. However, if the two answers are not next to each other, the auditor must read the notes from the interviewers. If there are no related notes, the auditor must consider the answer as missing.

All data were double entered with EPI DATA 2.1a (EpiData Association Odense, Denmark). The final dataset was converted into SPSS format. Data were analyzed primarily with SPSS version 11.0 (SPSS, USA) while confirmatory factor analysis was performed with SPSS Amos 4.0. Demographic data were assessed with descriptive statistics. Reliability assessment was conducted between interviewers. Twenty-four of the 25 interviewers were divided into 12 pairs, two in one pair. A pair of interviewers questioned a participant and it was ensured that they could not see each other's records. One of them was in charge of the investigation, while the other recorded the answers only. It was decided randomly who would be in charge. Inter-interviewer reliability was performed by intra-class correlation coefficient (ICC) analysis. Cronbach's α statistics were used to assess the internal consistency reliability of the HSTCM, in which values greater than 0.6 were considered evidence of internal consistency [27]. Unequal-length *Spearman-Brown prophecy coefficient *was used to obtain the split-half reliability coefficient [28].

Using input from both the HSTCM focus groups and Chinese medicine practitioners, we performed the face and content validation during the design phase of the HSTCM [21] to assess how well the HSTCM measured the concepts. The HSTCM was further verified after the design phase with input from healthy participants and inpatients of Chinese medicine hospitals to ensure the questions' relevance.

Convergent validation of the HSTCM was assessed with Spearman's rho correlation coefficients between the scores of the HSTCM and WHOQOL-BREF. An absolute value within the range of 0.4–0.8 suggests that the convergent validation is appropriate [23].

To study the ability of the domain and overall HSTCM scores in discriminating groups of participants of possibly different health status, we divided the participants into three groups according to their self-reported general health status, self-reported health status change and degree of medical needs. The mean, standard deviation (SD) and 95% confidence interval (95%CI) of domain and overall HSTCM scores of each group were calculated. A lack of overlapping between the ranges of 95%CI of any two groups suggests that the domain or the HSTCM can distinguish these groups.

Receiver operating characteristic (ROC) curves [29,30] were plotted for the HSTCM and its three domains. The area under the curve (AUC) was used as an indicator of the ability of the instrument to differentiate participants with or without good self-reported general health status. An AUC of greater than 0.50 indicates a discriminative accuracy to be greater than chance. A perfect discriminative ability would have an AUC of 1.0.

Principal component exploratory factor analysis with Equamax rotation was carried out to assess the underlying structure of the instrument among a set of questionnaire items. Confirmatory factor analysis was further performed to examine whether the data fit the applied model. Goodness of fit of the model was considered to be acceptable if root mean square error of approximation (RMSEA) is less than 0.08. Goodness of fit is good if RMSEA is less than 0.05[31]. Comparative fit index (CFI) of greater than 0.95 also indicates a satisfactory goodness of fit [32]. Values are considered statistically significant if *P *< 0.05.

## Results

### Range of responses

**Figure 1:**
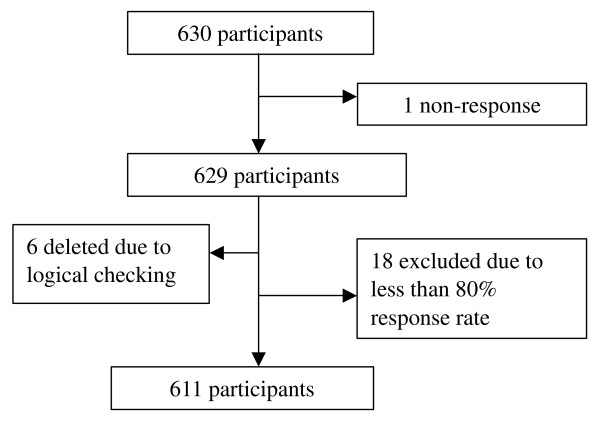
**Participant selection process**.

From December 2006 to February 2007, 630 participants as a convenience sample were recruited under 11 settings in the Liwan community in Guangzhou, China. Six hundred and twenty-nine participants returned both the HSTCM and WHOQOL-BREF questionnaires, giving an overall response rate of 99.8%. Six of the 629 questionnaires were excluded for not meeting the criteria of logical checking. Among the remaining 623 questionnaires, 611 responded to 80% or more of the items of both questionnaires (Figure 1). Missing values of the items were replaced in accordance with the series mean method.

### Demographics

Among the 611 participants, 58% were female and the mean age was 49 years. And 35.8% had completed high school and 15.9% had completed university or above. All participants were recruited under 11 settings of the Liwan community (Table 1). Among all demographic questionnaires, 66.9% were self-administered while 8.7% and 19.6% were interviewer-assisted and interviewer-administered respectively.

**Table 1:** Demographics of the participants

	No	Individual percentage (%)
Settings of the Liwan community		
Dahua	75	12.3
Fulixi	35	5.7
Fulidong	46	7.5
Huicheng	88	14.4
Wenchang flower garden	46	7.5
Fengyuanbei	50	8.2
Community Service centre	11	1.8
Majichong	100	16.4
Huagui	64	10.5
Houfu	53	8.7
Dade	41	6.8
Missing value	2	0.3
Age		
18–44	245	40.1
45–64	218	35.7
65 and older	145	23.7
Missing value	3	0.5
Gender		
Female	356	58.3
Male	252	41.2
Missing value	5	0.5
Education		
Elementary school	139	22.7
Junior high school	145	23.7
High school	219	35.8
Junior college and above	97	15.9
Missing value	11	1.8
Interview method		
Self-administered	409	66.9
Interviewer-assisted	53	8.7
Interviewer-administered	120	19.6
Missing value	29	4.7

### Reliability

#### Interviewer reliability

The overall correlation between interviewers in the HSTCM was 0.99 (95%CI: 0.996–0.999). The correlation coefficient of the PFNE domain was 0.99 (95%CI: 0.995–0.999), the SP domain 0.99 (95%CI: 0.996–0.999) and the SE domain 0.99 (95%CI: 0.996–0.999). All the intra-class correlation coefficients (for both the total scale and its three domains) were greater than 0.95 (the lower level of 95%CI > 0.90), suggesting that the inter-interviewer reliability was satisfactory.

#### Internal reliability

We calculated the Spearman-Brown split-half reliability coefficient to examine the split-half reliability. The coefficient was 0.81, indicating a good split-half reliability of the HSTCM.

Internal reliability of the HSTCM and its three domains were estimated with the Cronbach's α coefficient (Table 2).

**Table 2:** Cronbach's α coefficient of the HSTCM domains and facets

	Cronbach's α coefficient	No of items
HSTCM	0.93	45
1. PFNE	0.89	18
PF	0.89	9
VOC	0.75	2
SU	0.70	2
ANE	0.75	5
2. SP	0.90	14
CC	0.88	6
SC	0.90	2
EG	0.83	4
BAT	0.68	2
3. SE	0.87	13
ACP	0.76	2
ANC	0.32	2
ADBS	0.89	9

The Cronbach's α coefficients of the HSTCM domains and the total score of the HSTCM were larger than 0.80. Except the ANC facet, of which the Cronbach's α coefficient was 0.32, the values of other facets were greater than 0.65.

### Validation

#### Convergent validation and discriminative validation

We carried out convergent validation by correlating the total score of the HSTCM with that of the WHOQOL-BREF. The Spearman' rho correlation coefficient (r) was -0.67 (*P *< 0.001). The three-domain scores showed fair to moderate correlation with the four-domain scores of the WHOQOL-BREF. All the correlations were statistically significant. The physical function domain showed moderate correlation with physical domain (r = -0.55, *P *< 0.001) and had fair correlation between the psychological domain (r = -0.41, *P *< 0.001), social domain (r = -0.37, *P *< 0.001) and environmental domain (r = -0.31, *P *< 0.001) of the WHOQOL-BREF. The spirit domain showed higher correlation with the physical domain (r = -0.71, *P *< 0.001), psychological domain (r = -0.70, *P *< 0.001) and social domain (r = -0.53, *P *< 0.001) of the WHOQOL-BREF and fair correlation with the environmental domain (r = -0.47, *P *< 0.001). The social and environmental domain showed only fair correlation with all the four domains with correlation coefficient ranging from -0.32 (*P *< 0.001) in the social and environmental domain to -0.49 (*P *< 0.001) in the physical domain of the WHOQOL-BREF. The overall HSTCM score displayed significant correlation with all four domains of the WHOQOL-BREF. The correlation was moderate with the physical (r = -0.68, *P *< 0.001) and psychological domain (r = -0.62, *P *< 0.001) and fair with the social (r = -0.47) and environmental domain (r = -0.43, *P *< 0.001) of the WHOQOL-BREF (Table 3).

**Table 3:** Correlation between the HSTCM and WHOQOL-BREF domains

HSTCM domains	WHOQOL-BREF domains			
	Physical	Psychological	Social	Environmental
PFNE	-0.55	-0.41	-0.37	-0.31
SP	-0.71	-0.70	-0.53	-0.47
SE	-0.49	-0.47	-0.32	-0.32
Overall HSTCM	-0.68	-0.62	-0.47	-0.43

All correlations are significant at the 0.001 level (2-tailed).

In the HSTCM, a higher score means poorer health; in the WHOQOL-BREF, a higher value means better health.

#### Discriminative validation

The mean, standard deviation (SD), 95%CI of each group of the HSTCM and its domains were calculated (Tables 4 and 5). No overlapping of the 95%CI was found between the participants with good self-reported health status and those with poor self-reported health status in all three domain scores and the overall HSTCM scores. The same results were found between participants with better self-reported health status change and those with worse health status, and between participants with 'not at all/a little degree of medical needs' and those with 'very much/great degree of medical needs'. The overall HSTCM scores and the three domain scores differentiated all three levels of the self-reported health status indicators as well as discriminated the participants with 'not at all/a little degree of medical needs' from those with 'moderate needs'. However, the three domain scores and the overall HSTCM scores did not distinguish the participants with 'moderate medical needs' from those with 'very much/great medical needs'. Moreover, the scores did not distinguish the participants with 'better health statuses' from those with 'no change'.

**Table 4:** Discriminative ability of the HSTCM in terms of levels of health status

		Scores of the HSTCM		
	No.	Mean	SD	95%CI
1. Self-reported general health status				
				
Good/very good	268	90.18	18.56	87.86–92.69
Neither poor nor good	263	114.46	19.16	112.46–117.40
Poor/very poor	78	132.92	21.50	128.03–137.80
Missing value	2	-	-	-
				
2. Self-reported health status change				
				
Better	102	93.92	24.21	88.92–99.82
No change	283	102.17	22.72	99.73–105.44
Worse	226	116.25	22.50	113.54–119.76
Missing value	0	-	-	-
				
3. Degree of medical needs				
				
Not at all/a little	331	97.56	21.33	94.47–99.42
A moderate amount	189	115.97	22.17	113.36–119.86
A great amount	88	117.20	26.98	113.44–125.45
Missing value	3	-	-	-

Self-reported general health status is item 21 of the HSTCM; self-reported health status change is item 22 of the HSTCM; degree of medical needs is item 4 of the WHOQOL-BREF.

**Table 5:** Discriminate ability of the domains in terms of levels of health status

	No	Scores of PFNE			Scores of SP			Scores of SE		
		Mean	SD	95%CI	Mean	SD	95%CI	Mean	SD	95%CI
1. Self-reported general health status										
Good/very good	268	34.8	8.80	33.5–35.8	27.9	6.85	27.3–29.0	27.5	7.59	26.5–28.5
Neither poor nor good	263	45.3	9.82	44.4–46.9	36.6	6.60	35.9–37.6	32.6	7.68	31.5–33.5
Poor/very poor	78	51.2	11.77	49.1–54.6	44.4	6.78	42.9–45.9	36.5	8.09	34.8–38.6
Missing value	2	-	-	-	-	-	-	-	-	-
										
2. Self-reported health status change										
Better	102	38.0	11.15	35.4–40.3	27.0	8.46	25.6–29.4	28.9	8.35	27.2–30.9
No change	283	39.4	11.07	38.3–41.1	32.9	7.87	32.1–34.0	29.8	8.03	28.8–30.8
Worse	226	45.5	10.90	44.2–47.3	37.8	7.79	36.8–39.0	33.0	8.26	31.8–34.1
Missing value	0	-	-	-	-	-	-	-	-	-
										
3. Degree of medical needs										
Not at all/a little	331	37.8	9.61	36.3–38.6	30.8	8.04	30.0–31.7	29.0	7.87	27.8–29.6
A moderate amount	189	46.0	11.25	44.8–48.0	37.2	7.60	36.3–38.6	32.7	7.94	31.6–33.9
A great amount	88	45.7	13.30	43.6–49.6	37.4	9.56	36.1–40.3	34.1	8.95	32.6–36.6
Missing value	3	-	-	-	-	-	-	-	-	-

Self-reported general health status is item 21 of the HSTCM; self-reported health status change is item 22 of the HSTCM; degree of medical needs is item 4 of the WHOQOL-BREF.

PFNE: physical function under natural environment domain

SP: spirit domain

SE: social and environment domain

**Figure 2:**
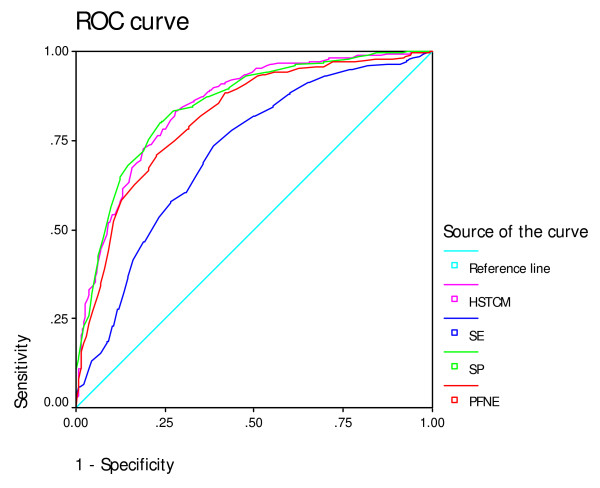
**ROC curves for the HSTCM and its domains in discriminating performance between participants with good self-reported general health status and those without**. HSTCM: Health Scale of Traditional Chinese Medicine; SE: social and environment domain; SP: spirit domain; PFNE: physical function under natural environment domain.

ROC curves showed the discriminate validation of the instrument (Figure 2). The AUC for the HSTCM and its domains were high and all the lower levels of 95%CI were greater than 0.5 (Table 6), suggesting that the instrument discriminated the participants in terms of self-reported health status.

**Table 6:** Areas under the ROC curves for the HSTCM and its domains in discriminate performance between participants with and without good self-reported general health status

Variable	Area under ROC curve	SE	Significant level	95%CI	
				Lower	Upper
PFNE	0.81	0.02	< 0.01	0.78	0.85
SP	0.85	0.02	< 0.01	0.82	0.88
SE	0.71	0.02	< 0.01	0.67	0.76
HSTCM	0.85	0.02	< 0.01	0.82	0.88

Null hypothesis: true area = 0.5 (Smaller test result indicates more positive test.)

Nonparametric distribution (95%CI)

#### Construct validation

##### Exploratory factor analysis

The previous version of the HSTCM [21] did not include appetite and sleep. As appetite and sleep are often used in diagnosis and outcome assessment, the HSTCM focus group decided to include them as additional items of the HSTCM, bringing the total number of items to 47. However, these two items were not included in the validation analysis. By exploratory factor analysis, 45 items were summed up to nine factors (Table 7).

**Table 7:** Exploratory factor analysis of the nine facets of the HSTCM

	1	2	3	4	5	6	7	8	9
1. PF									
Palpitation (T14)	0.753								
Chest distress (T15)	0.752								
Disorder of the taste (T18)	0.709								
Tinnitus (T20)	0.677								
Thirst (T17)	0.666								
Dizziness (T_13)	0.652								
Pain (T_16)	0.652								
Disorder of perspiration (T12)	0.638								
Gastrointestinal disorder (T19)	0.618								
2. SDSC, CC									
Satisfaction of appearance (T31)		0.781							
Satisfaction of body figure (T30)		0.774							
Concerning the future (T29)		0.749							
Meaning of life (T32)		0.736							
Contented sensation of work (T28)		0.703							
Vivid (T26)		0.689							
Joy (T25)		0.678							
Confidence (T27)		0.674							
3. ADBS									
Great concern (T39)			0.732						
Upset (T41)			0.721						
Afraid (T38)			0.696						
Tension and tired (T42)			0.683						
Scare (T40)			0.669						
Worry (T36)			0.599						
Hesitate (T43)			0.595						
Insomnia (T37)			0.579						
Annoyance (T35)			0.531						
4. EG									
Self-reported health change (T22)				0.749					
Energy (T23)				0.748					
Self-reported general health(T21)				0.680					
Physical strength (T24)				0.675					
5. ANE									
Cold weather (T2)					0.697				
Weather change (T1)					0.639				
Hot weather (T3)					0.615				
Season change (T4)					0.612				
Circumstance of temperature difference (T5)					0.586				
6. VOC, SU									
Urine (T10)						0.677			
Stool (T11)						0.596			
Voice (T7)						0.630			
Voice change (T6)						0.452			
7. ACP									
Nervous (T44)							0.773		
Adjustment disorders (T45)							0.753		
8. BAT									
Activities of daily life (T33)								0.686	
Focus attentions (T34)								0.562	
9. ANC									
Anti-disturbance capability (T46)									0.726
Noisy environment (T47)									0.466

Extraction method: principal component analysis. Equamax rotation with Kaiser Normalization, sorted by size, factor loading < 0.4 suppressed

Result of the Kaiser-Meyer-Olkin (KMO) measure of sampling adequacy test was 0.93, suggesting that the data were suitable for factor analysis [33]. The nine facets explained about 60.8% of the total variance of the data. The constructs of facet were very similar to those of the previous version. Only SC and CC, belonging to two facets, emerged in the same factor; so did VOC and SU. SC and CC were the facets of the SP domain, while VOC and SU belonged to the PFNE domain. These results showed that the constructs of facets were coherent with structures of the HSTCM domains.

We performed further exploratory factor analysis using scores of the nine facets resulting in three factors, which explains the total variance of 64.1% (Table 8).

**Table 8:** Exploratory factor analysis of the three domains of the HSTCM

	1	2	3
PF	0.764		
ANE	0.776		
VOC, SU	0.632		
SC, CC		0.814	
EG		0.618	
BAT		0.771	
ACP			0.771
ANC			0.755
ADBS			0.518

Extraction method: principal component analysis Equamax rotation with Kaiser Normalization, sorted by size, factor loading < 0.4 suppressed

##### Confirmatory factor analysis

**Figure 3:**
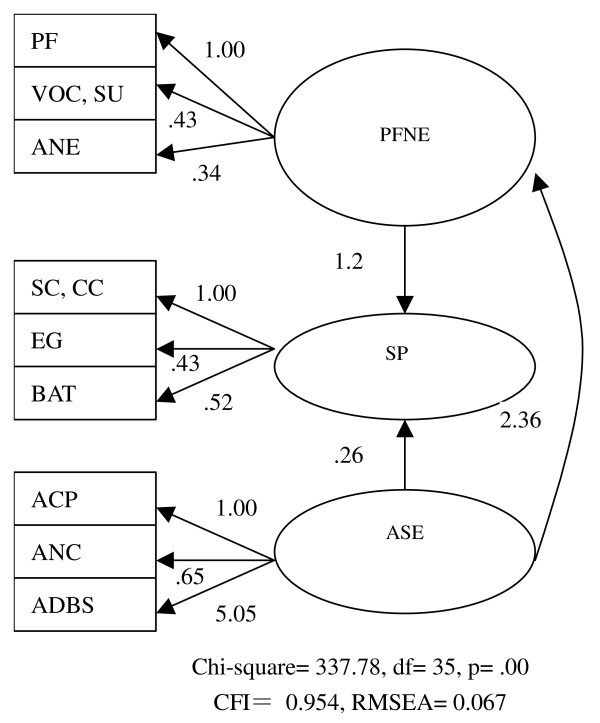
**Model of the confirmatory factor analysis between the HSTCM domains and facets**.

We constructed a model to carry out the confirmatory factor analysis (Figure 3). CFI of the model was above 0.90, while the RMSEA was less than 0.08. The goodness of fit was acceptable.

## Discussion

The properties of the HSTCM were tested in a group of participants in the Liwan community in Guangzhou, China. Measurement properties, internal reliability and inter-interviewer reliability of the HSTCM and its domains were satisfactory. The HSTCM had mild convergence with the WHOQOL-BREF (with a moderate correlation). The overall HSTCM scores and most of the domain scores discriminated groups of participants with known differences in health status. Confirmatory factor analysis showed a good structure fitness of the domains and overall structure.

In the reliability test, Cronbach's α was low in subscale ANC (0.32). As the items in the ANC facet met the selection criteria and Cronbach's α was considered to be acceptable on the whole scale and three domains' level; we may need further evidence before removing ANC from the scale.

In this study, we used the ROC analysis to evaluate the discriminate ability of the HSTCM. The AUC indicates that the HSTCM discriminate better than chance between groups with and without good self-reported health status. Therefore, the results of ROC analysis support the clinical validity of the HSTCM. However, this discriminate ability should not be confused with that of diagnostic instruments. Health status measures are not intended to be used at an individual patient level [34].

Construct validation is an ongoing process. The more frequently an instrument is used, the greater our confidence is in its validation [35]. Comparing the previous study [21] with the present one, we found that the construct validities of two independent samples were similar. Similar constructs in two independent studies strengthen the construct validation for the HSTCM. We may find some similar constructs in other health related QOL instruments based on Chinese medicine theory [16].

Items from SC and CC had similar meanings in terms of the HSTCM developmental theory, as did VOC and SU, which may be explained by the exterior-interior relations [36] between the large intestine meridian and lung meridian. The exterior-interior relations of the two meridians not only strengthen the connection between this specific pair of meridians, but also promote the large intestine/lung pair to coordinate with each other physiologically and influence each other pathologically. Items of the VOC facet are key indicators of the status of lung, while the SU facet contains an important predictor of the large intestine.

No test-retest reliability was conducted in the present study; however, results from the previous study suggest good test-retest reliability [22].

The attribute of 'responsiveness' is also considered as a component of a scale validation [37]. Based on the discriminative validation and measures of ROC, we think that the responsiveness of the scale is acceptable [38]. However, we did not estimate the parameter to compare the before-after changes caused by a given intervention, which means the inference may be limited.

In this study, participants were selected from a community by convenience sampling which is widely used in psychometric property testing, especially those of health scales [39-43]. However, a convenience sample is less representative than a random sample and may limit the generalizability of this study.

We did not use item response theory (IRT) in developing and evaluating the HSTCM because the advantage of the IRT methods over traditional methods was not clear to us [44]. However, we think that IRT methods can be used to refine the scale. Generalizability theory (GT) is another powerful tool in analyzing scale reliability with complicated sources of measurement biases. Further studies with IRT to analyze item characteristic of the instrument and with GT to evaluate the validation of the HSTCM are warranted.

## Conclusion

The HSTCM meets the basic requirements for a generic health scale and complements the existing health measurement scales for Chinese medicine health care.

## Abbreviations

HSTCM: Health Scale of Traditional Chinese Medicine; WHOQOL-BREF: World Health Organization Quality of Life Measure-Abbreviated Version; SF-36: MOS 36-Item Short Form; PRO: patient reported outcomes; SOP: standard operating procedure; iHSTCM: initial Health Scale of Traditional Chinese Medicine; ICC: intraclass correlation coefficient; PFNE: physical function under natural environment domain; SP: spirit domain; SE: social and environment domain; PF: physical functioning facet; VOC: voice facet; SU: stool and urine facet; ANE: adaptability of natural environment facet; CC: confidence and content facet; SC: self-confidence facet; EG: energy facet; BAT: basic ability of thinking facet; ACP: ability of communicating with people facet; ANC: adaptability of noisy condition facet; ADBS: ability of dealing with bad stimulations facet; ROC: receiver operating characteristic; AUC: area under curve; CFI: comparative fit index; RMSEA: root mean square error of approximation; IRT: item response theory; GT: generalizability theory.

## Competing interests

The authors declare that they have no competing interests.

## Authors' contributions

SLL and DRW conceived the study design, drafted the protocol and manuscript. DRW and LJZ arranged and conducted the training and investigation. DRW did data management and data analysis. XFG, ZHW, WXL and AHO were members of the HSTCM focus group; together with KJC they provided methodological insights. GQZ was in charge of the recruitment of the interviewers. All authors read and approved the final version of the manuscript.

## Supplementary Material

Additional file 1 **The items of the HSTCM in their original Chinese**. The HSTCM consists of three domains, namely physical function under natural environment (PFNE), spirit (SP) and social environment (SE), and 2 additional items. Click here for file
