# Supplementary material for: Further validation of the Health Scale of Traditional Chinese Medicine (HSTCM)
Source: Chin Med. 2009 Apr 30;4:8. doi: 10.1186/1749-8546-4-8 (PMC2684103; doi:10.1186/1749-8546-4-8)
Supplement: Additional file 1 — The items of the HSTCM in their original Chinese. The HSTCM consists of three domains, namely physical function under natural environment (PFNE), spirit (SP) and social environment (SE), and 2 additional items. [file 1749-8546-4-8-S1.doc]

Additional file 1: The items of the HSTCM in their original Chinese

| **Facet codes and names** | **Item No.** | **Items in original Chinese and English translations (note1)** | **Reverse polarity of the scale (note 2)** |
| --- | --- | --- | --- |
| **Physical Function under Natural Environment Domain** |  |  |  |
| HS1.1 ANE (Adaptability of Natural Environment) | 1 | 天气变化的时候，您会容易生病吗？ | No |
|  | 2 | 您在冬天怕冷吗？ | No |
|  | 3 | 您在夏天怕热吗？ | No |
|  | 4 | 季节的转变对您的健康有影响吗？ | No |
|  | 5 | 频繁出入温差大的地方（如有空调的地方），您会容易生病吗？ | No |
| HS1.2 VOC (Voice) | 6 | 您的声音和一年以前相比有变化吗？ | No |
|  | 7 | 最近一个月，您说话的声音对您的日常生活有影响吗？ | No |
| HS1.3 SU (Stool and Urine) | 10 | 最近一个月，您的小便正常吗？ | No |
|  | 11 | 最近一个月，您的大便正常吗？ | No |
| HS1.4 PF (Physical functioning) | 12 | 最近一个月，您会出现汗出异常的情况吗？（如出汗过多、出汗过少、睡觉时出汗等） | No |
|  | 12.1 | 汗出异常对您的日常生活影响如何？ |  |
|  | 13 | 最近一个月，您有头晕的感觉吗？ | No |
|  | 13.1 | 头晕的感觉对您的日常生活影响如何？ |  |
|  | 14 | 最近一个月，您有心慌／心跳的感觉吗？ | No |
|  | 14.1 | 心慌／心跳的感觉对您的日常生活影响如何？ |  |
|  | 15 | 最近一个月，您有胸闷／胸胁不适的感觉吗？ | No |
|  | 15.1 | 胸闷/胸胁不适的感觉对您的日常生活影响如何？ |  |
|  | 16 | 最近一个月，您有疼痛的感觉吗？ | No |
|  | 16.1 | 疼痛对您的日常生活影响如何？ |  |
|  | 17 | 最近一个月，您觉得口渴吗？ | No |
|  | 17.1 | 口渴对您的日常生活影响如何？ |  |
|  | 18 | 最近一个月，您觉得口中有让您不舒服的味道吗？（如口臭、口苦、口淡、口甜、口酸、口中粘腻等） | No |
|  | 18.1 | 这些不舒服的味道对您的日常生活影响如何？ |  |
|  | 19 | 最近一个月，您有胃肠不适的感觉吗？（如胃胀、腹胀） | No |
|  | 19.1 | 这种感觉对您的日常生活影响如何？ |  |
|  | 20 | 最近一个月，您有耳鸣吗？ | No |
|  | 20.1 | 耳鸣对您的日常生活影响如何？ |  |
| **Spirit domain** |  |  |  |
| HS2.1 EG (Energy) | 21 | 总的来说，您认为自己的健康状况如何？ | Yes |
|  | 22 | 与一年前相比，您目前的健康状况如何？ | Yes |
|  | 23 | 最近一个月，您觉得精力充沛吗？ | Yes |
|  | 24 | 最近一个月，您有足够的体力进行一般的运动吗？ | Yes |
| HS2.2CC(Confidence and content) | 25 | 您现在生活得快乐吗？ | No |
|  | 26 | 生活中，您常能发觉许多开心的事吗？ | No |
|  | 27 | 您感到自己是有用的或/和不可缺少的人吗？ | No |
|  | 28 | 您能从您做的工作和/或家务事中得到满足吗？ | No |
|  | 29 | 您觉得未来会好吗？ | No |
|  | 32 | 对于您来说，生活有意义吗？ | No |
| HS2.3SC(Self-confidence) | 30 | 您对自己的外形满意吗？ | No |
|  | 31 | 您对您的相貌满意吗？ | No |
| HS2.4BAT(Basic ability of thinking) | 33 | 最近一个月，您能独自去到较远的地方吗？ | No |
|  | 34 | 最近一个月，您能持续一小时以上注意力集中地干一件事吗？ | No |
| **Social Environment domain** |  |  |  |
| HS3.1ADBS(Ability of dealing with bad stimulations) | 35 | 最近一个月，您会因为一点儿小事而烦恼很久吗？ | No |
|  | 36 | 最近一个月，您会为可能发生的事情而操不必要的心吗？ | No |
|  | 37 | 在有事情挂着的时候，您会失眠吗？ | No |
|  | 38 | 最近一个月，您会无缘无故地为一些不现实的东西担心吗？ | No |
|  | 39 | 多数时间里，您都会为某些人／事感到忧心忡忡吗？ | No |
|  | 40 | 最近一个月，您会不会比别人容易受惊吓？ | No |
|  | 41 | 您很容易因为一些意想不到的事情而吃惊吗？ | No |
|  | 42 | 最近一个月，您会因为一点儿小事而感到紧张和疲惫吗？ | No |
|  | 43 | 最近一个月，您做事情总是会犹豫不决吗？ | No |
| HS3.2ACP(Ability of communicating with people) | 44 | 当众讲话会使您紧张吗？ | No |
|  | 45 | 陌生的人或场合会使您感到紧张或害怕吗？ | No |
| HS3.3ANC(Adaptability of Noisy Condition) | 46 | 如果周围很吵闹，您可以集中注意力吗？ | Yes |
|  | 47 | 嘈杂的环境中，您会感到明显的不适吗？ | No |
| Additional item 1 | 8 | 最近一个月，您胃口好吗？ | No |
| Additional item 2 | 9 | 最近一个月，您睡得好吗？ | No |

Note 1. Reverse polarity of the scale are need for items marked with “yes”, i.e. change score 1 to 5, 2 to 4, 3 to 3, 4 to 2 and 5 to 1.
